# Effects of Dexmedetomidine on Delirium in Trauma Intensive Care Unit (ICU) Patients: A Retrospective Cohort Study

**DOI:** 10.7759/cureus.83573

**Published:** 2025-05-06

**Authors:** Yahia Homsi, Wesley Kafka, Aous Jarrouj, Tiffany M Lasky, Rommy P Obeid, Maria P Mace, Damayanti Samanta

**Affiliations:** 1 Behavioral Medicine and Psychiatry, Charleston Area Medical Center, Charleston, USA; 2 Pharmacy/Surgical Trauma ICU, Charleston Area Medical Center, Charleston, USA; 3 Center for Health Services and Outcomes Research, Charleston Area Medical Center Institute for Academic Medicine, Charleston, USA; 4 Critical Care Surgery, Charleston Area Medical Center, Charleston, USA; 5 Medicine, West Virginia School of Osteopathic Medicine, Charleston, USA

**Keywords:** delirium, dexmedetomidine, risk factors for delirium, sedation, surgical-trauma icu

## Abstract

Background

While some studies suggest that dexmedetomidine is a strong prophylactic against delirium, there is a lack of compelling evidence supporting its use in critically ill patients who have suffered traumatic injuries requiring treatment in intensive care settings. The primary objective of this study was to evaluate the effect of dexmedetomidine on the incidence of delirium in trauma ICU patients. Given the absence of a significant association, a secondary analysis was conducted to identify independent predictors of delirium.

Methods

A retrospective cohort study was conducted among adult patients with traumatic injuries admitted to the surgical-trauma ICU at a level 1 trauma center between 2017 and 2021. Level 1 trauma centers serve as regional referral centers, often managing the most severe and complex trauma cases. Patients were categorized into the dexmedetomidine-based sedation group (receiving dexmedetomidine along with other concomitant sedatives) and the non-dexmedetomidine-based sedation group (receiving other sedative agents, excluding dexmedetomidine).

Results

Of the 272 patients included in the study, 163 (60%) were in the dexmedetomidine-based sedation group. The incidence of delirium was comparable between dexmedetomidine-based and non-dexmedetomidine-based sedation groups (13.0% vs. 9.2%, p = 0.33). The risk of delirium was approximately threefold higher in patients with a pre-existing psychiatric illness (OR = 2.65, 95% CI 1.14-6.30, p = 0.02) and almost fourfold in patients with exposure to benzodiazepine (OR = 3.90, 95% CI 1.36-11.72, p = 0.02).

Conclusions

This study adds to the existing literature by presenting data on the incidence of delirium among trauma patients and assessing how dexmedetomidine affects its prevalence. The findings align with the current body of research, highlighting that pre-existing psychiatric conditions and benzodiazepine use are recognized risk factors for delirium in the trauma ICU population. Nevertheless, dexmedetomidine administration was not found to significantly influence the likelihood of developing delirium in these patients.

## Introduction

Delirium is an acute disorder of consciousness and cognition, prevalent in critically ill ICU patients, with an incidence of 20%-50% in non-ventilated and 60%-80% in mechanically ventilated patients [1]. The Richmond Agitation and Sedation Scale (RASS) and the Confusion Assessment Method for the ICU (CAM-ICU) are commonly used for delirium assessment [2,3]. Despite the availability of these tools, delirium, particularly in its hypoactive form, remains underdiagnosed. Delirium is associated with increased ICU and length of hospital stay, higher costs, long-term cognitive impairment, and increased mortality [4].

Current delirium prevention strategies emphasize non-pharmacologic interventions, such as early mobility and physical therapy, routine delirium screening and early detection, and sleep promotion. The implementation of light sedation protocols, daily sedation interruption, and the avoidance of benzodiazepines have also shown a positive impact on the incidence of delirium [1]. The main goal of sedation in the intensive care setting is to keep the patient calm, cooperative, easy to arouse, and able to communicate their needs, especially for analgesia. Guidelines recommend the use of dexmedetomidine, propofol, and benzodiazepines for sedation in the ICU and propose that nonbenzodiazepine strategies may be favored over benzodiazepine agents [5]. 

Dexmedetomidine is a selective α2-adrenoceptor agonist with sedative, analgesic, and opioid-sparing effects, suitable for both short- and long-term sedation in critically ill patients [6]. It allows for easier arousal and better communication compared to midazolam or propofol [7] and induces minimal respiratory depression, even at higher doses. Although it can cause bradycardia due to the decrease in sympathetic tone and hypotension due to the predominant vasodilator effects of the central α-2A receptors, these adverse effects are manageable with atropine and vasoactive agents [8]. Its favorable physiological profile has led to an expanded clinical application.

While dexmedetomidine may produce less delirium than other sedatives or even prevent delirium, its efficacy for sedation in critically ill trauma patients remains uncertain [8]. Trauma patients in the surgical-trauma intensive care unit (STICU) are often frail, hemodynamically unstable, and suffering from multiple injuries, factors that may influence delirium differently compared to patients in medical or other surgical ICUs. This study aims to evaluate the impact of dexmedetomidine on delirium incidence in trauma patients and identify predictors of delirium in this population.

## Materials and methods

This retrospective cohort study was conducted at the Charleston Area Medical Center's Level 1 trauma center located in Charleston, WV, USA. Patients included in this study were aged >18 years and admitted to STICU between January 1, 2017, and December 31, 2021. Level 1 trauma centers provide the highest level of surgical care to trauma patients, equipped with 24/7 in-house coverage by trauma surgeons, and immediate availability of specialists in orthopedics, neurosurgery, anesthesiology, emergency medicine, radiology, and critical care. These centers also serve as regional referral centers, often managing the most severe and complex trauma cases. Patients were categorized into two cohorts: the study group, receiving dexmedetomidine along with other concomitant sedatives, hereafter referred to as the dexmedetomidine-based sedation group, and the control group, receiving other sedative agents (benzodiazepines, propofol, and opioids, excluding dexmedetomidine), hereafter referred to as the non-dexmedetomidine-based sedation group. Patients were excluded if they received ketamine during their STICU stay or had a documented history of schizophrenia or schizoaffective disorder. Additionally, patients who stayed ≤24 hours in the STICU were excluded, as delirium incidence could not be adequately assessed in this group.

Study data were obtained from the institutional trauma registry and electronic medical records (EMRs). The institutional review board approved the study (IRB# 21-810). Delirium incidence was assessed through EMR review, along with data on home medications (antipsychotics, antidepressants, benzodiazepines), hospital medications (dexmedetomidine, benzodiazepines, propofol, opioids), respiratory status (intubation vs. no intubation), and hypotension (systolic blood pressure < 90 mmHg or mean arterial pressure < 65 mmHg) or bradycardia (heart rate < 60 bpm) within 24 hours of dexmedetomidine administration. Baseline characteristics, including age, sex, injury severity score (ISS), pre-existing conditions, and traumatic brain injury (TBI) diagnosis, were retrieved from the trauma registry.

Descriptive analysis was conducted for each variable in this study. Means and standard deviations or medians and interquartile ranges for continuous variables were reported. For categorical variables, proportions and frequencies were computed. To assess statistically significant differences between the two cohorts, t-tests were conducted for continuous variables. Categorical variables were compared using chi-square test. Logistic regression was performed to determine predictors for delirium with variables that were significant on univariate analysis. All comparisons were performed at a level of significance of p ≤ 0.05. Analysis was performed using IBM SPSS Statistics for Windows, Version 22.0 (Released 2013; IBM Corp., Armonk, NY, USA).

## Results

Of the 272 patients included in this study, 163 patients (60%) were in the dexmedetomidine-based sedation group, and the remaining 109 patients (40%) were in the non-dexmedetomidine-based sedation group. Patients in both these groups were comparable in age, sex, ISS, pre-existing conditions, and TBI incidence. The only significant difference was a higher intubation rate in the dexmedetomidine-based sedation group (79.1% vs. 19.3%, p < 0.001) (Table 1). Intake of antipsychotics, antidepressants, and benzodiazepines at home prior to admission to the STICU was also comparable between the two groups (Table 2).

**Table 1:** Comparison of patient characteristics between the dexmedetomidine and non-dexmedetomidine groups ^*^Indicates statistical significance at p < 0.05. Statistical analyses were performed using t-tests for continuous variables and chi-square tests for categorical variables.

Characteristic	Dexmedetomidine (N = 163)	Non-dexmedetomidine (N = 109)	p-value
Age, mean ± SD	56.77 ± 20.36	56.80 ± 21.57	0.99
Sex (male), n (%)	121 (74.2%)	79 (71.8%)	0.65
Injury severity score, mean ± SD	16.19 ± 8.36	15.73 ± 8.20	0.65
Comorbidities, n (%)			
Alcohol and substance use	53 (32.5%)	26 (23.9%)	0.12
Cardiovascular disease	90 (55.2%)	62 (56.0%)	0.90
Diabetes mellitus	36 (22.1%)	24 (21.1%)	0.84
Neurologic disease	5 (3.1%)	3 (2.8%)	0.88
Psychiatric illness	32 (19.6%)	18 (16.5%)	0.51
Traumatic brain injury, n (%)	93 (57.1%)	69 (63.3%)	0.30
Intubation, n (%)	129 (79.1%)	21 (19.3%)	<0.001*

**Table 2:** Comparison of home medications between the dexmedetomidine and non-dexmedetomidine groups ^a^Some patients were taking more than one antipsychotic medication. ^b^Some patients were taking multiple antidepressant medications. Statistical analyses were performed using chi-square tests.

Medications	Dexmedetomidine (N = 163)	Non-dexmedetomidine (N = 109)	p-value
Home antipsychotic, n (%)^a^	21 (12.9%)	9 (8.3%)	0.23
Amitriptyline	1		
Aripiprazole	1	1	
Latuda	1		
Olanzapine	6		
Paliperidone		1	
Quetiapine	5	2	
Risperidone	2	1	
Ziprasidone	1		
Zyprexa	4	4	
Loxapine	1		
Home antidepressant, n (%)^b^	45 (27.6%)	27 (24.8%)	0.60
Venlafaxine		1	
Amitriptyline		1	
Bupropion	5	4	
Buspar		1	
Carbamazepine		1	
Citalopram	3	2	
Duloxetine	3		
Escitalopram	4	2	
Fluoxetine	4	4	
Mirtazapine	3	3	
Nortriptyline	1		
Paroxetine	5		
Paxil		1	
Prozac		1	
Sertraline	10	3	
Trazadone	10	5	
Venlafaxine	3	3	
Vilazodone		1	
Home benzodiazepine, n (%)	12 (7.4%)	3 (2.8%)	0.10
Lorazepam	12	3	

After admission to the STICU, the incidence of delirium was similar between the dexmedetomidine-based and the non-dexmedetomidine-based sedation groups (13.0% vs. 9.2%, p = 0.33). Patients in the dexmedetomidine-based sedation group were significantly more likely to have received a second sedating agent, such as a benzodiazepine or an opioid, while in the STICU. The proportion of patients receiving benzodiazepines (83.4% vs. 39.4%, p < 0.001), opioids (94.5% vs. 80.7%, p < 0.001), or propofol (65.0% vs. 16.5%, p < 0.001) was significantly higher in the dexmedetomidine-based sedation group as compared with the non-dexmedetomidine-based sedation group (Table 3). The median ICU stay was significantly longer for patients who received dexmedetomidine (8 days vs. 3 days, p < 0.001).

**Table 3:** Comparison of exposure to medications in the surgical-trauma intensive care unit between the dexmedetomidine and non-dexmedetomidine groups ^*^Indicates statistical significance at p < 0.05. Statistical analyses were performed using chi-square tests.

Medications	Dexmedetomidine (N = 163)	Non-dexmedetomidine (N = 109)	p-value
Benzodiazepine, n (%)	136 (83.4%)	43 (39.4%)	<0.001^*^
Lorazepam	104 (63.8%)	27 (24.8%)	<0.001^*^
Midazolam	89 (54.6%)	25 (22.9%)	<0.001^*^
Opioids, n (%)	154 (94.5%)	88 (80.7%)	<0.001^*^
Fentanyl	133 (81.6%)	50 (45.9%)	<0.001^*^
Hydromorphone	127 (77.9%)	51 (46.8%)	<0.001^*^
Morphine	42 (25.8%)	24 (22.0%)	0.48
Oxycodone	95 (58.3%)	38 (34.9%)	<0.001^*^
Hydrocodone	0 (0.0%)	7 (6.4%)	<0.01^*^
Propofol, n (%)	106 (65.0%)	18 (16.5%)	<0.001^*^

Dexmedetomidine is known to affect hemodynamic changes, which can be exacerbated by opioid use. Hemodynamic data detailing hypotension or bradycardia within 24 hours of dexmedetomidine administration was available for 121 patients in the dexmedetomidine-based sedation group. Of those patients, 68 (56.2%) had hypotension and 34 (24.6%) had bradycardia.

Since dexmedetomidine administration was not found to be associated with the incidence of delirium in this cohort of patients with traumatic injuries requiring STICU care, further analyses were conducted to examine independent risk factors for delirium in the cohort. The risk of delirium was nearly threefold higher in patients with a pre-existing psychiatric illness (OR 2.65, 95% CI 1.14-6.30, p = 0.02) and nearly fourfold higher in those exposed to benzodiazepines (OR 3.90, 95% CI 1.36-11.72, p = 0.01). Administration of dexmedetomidine did not significantly alter the risk of developing delirium (OR 0.76, 95% CI 0.29-1.96, p = 0.57) (Table 4).

**Table 4:** Independent predictors of delirium in surgical-trauma ICU patients ^*^Indicates statistical significance at p < 0.05. Statistical analyses were performed using logistic regression.

Factors significant in univariate analysis	Odds ratio	95%CI	p-value
Age	1.03	1.01-1.06	<0.01^*^
Dexmedetomidine	0.76	0.29-1.96	0.57
Comorbidities			
Cardiovascular disease	1.15	0.37-3.57	0.80
Diabetes mellitus	1.60	0.66-3.84	0.29
Psychiatric illness	2.65	1.14-6.30	0.02^*^
Home antipsychotic use	2.11	0.74-6.03	0.16
Exposure to benzodiazepine in the surgical-trauma ICU	3.90	1.36-11.72	0.01^*^

## Discussion

This study was novel in assessing the safety and efficacy of dexmedetomidine in patients with traumatic injuries admitted to STICUs. We found that dexmedetomidine was not effective in the treatment or prevention of delirium in patients with traumatic injuries requiring ICU care, as the incidence of delirium was comparable in the dexmedetomidine-based sedation group and the non-dexmedetomidine-based sedation group. Pre-existing psychiatric illness and exposure to benzodiazepines were identified as risk factors for delirium in these patients. To our knowledge, dexmedetomidine use has only been studied in critically ill patients cared for in the surgical ICU postoperatively or in the medical ICU. This study provides important insights into how to best care for patients admitted to the ICU for traumatic injuries.

Our finding that the incidence of delirium was similar between the dexmedetomidine-based and non-dexmedetomidine-based sedation groups contrasts with existing literature, which suggests a positive impact of dexmedetomidine in preventing delirium [9-11]. However, most supporting studies were conducted in non-trauma patients. For instance, a 2016 randomized trial with 350 patients per arm demonstrated that low-dose dexmedetomidine (0.1 μg/kg/hour) reduced postoperative delirium in older ICU patients, both intubated and non-intubated [12]. A 2019 meta-analysis of 25 randomized controlled trials (RCTs) with 3240 patients also found significant reductions in delirium and agitation in ICU patients using dexmedetomidine, primarily in surgical ICU settings [9]. Similarly, a 2020 meta-analysis of six RCTs and two cohort studies found lower delirium incidence with dexmedetomidine compared to propofol in patients ≥60 years in ICU settings [10]. Most recently, a meta-analysis of 77 RCTs in mechanically ventilated patients across surgical and medical ICUs showed a reduced risk of delirium with dexmedetomidine compared to other sedatives [11].

Contrary to other studies, we observed a significantly longer median ICU stay in trauma patients in the dexmedetomidine-based sedation group (eight days vs. three days). For instance, Su et al. [12] found that dexmedetomidine reduced ICU (20.9 hours vs. 21.5 hours) and hospital stays (10 days vs. 11 days) in post-surgical ICU patients. A 2019 meta-analysis also reported shortened ICU and hospital stays and reduced mechanical ventilation duration with dexmedetomidine [9]. However, a 2020 meta-analysis found no significant differences in ICU stay or mechanical ventilation duration in medical and surgical ICU patients [10], and a 2022 meta-analysis reported low certainty regarding reductions in ICU stay and mechanical ventilation with dexmedetomidine [11].

Reports on drug-associated adverse events, particularly hypotension and bradycardia, following dexmedetomidine administration have been mixed. A low-dose infusion (0.1 μg/kg/hour) did not increase bradycardia or hypotension incidence and also did not produce significant sedation [12]. Conversely, a loading dose increased bradycardia and hypotension incidence without affecting mortality [9]. In this study, over half of the patients in the dexmedetomidine-based sedation group experienced hypotension, and one-quarter experienced bradycardia, highlighting the importance of monitoring for hemodynamic complications following dexmedetomidine administration.

We found that benzodiazepine administration in the STICU and pre-existing psychiatric illness were independent risk factors for delirium, consistent with existing literature. A 2005 prospective study of 100 surgical and trauma ICU patients found a 2.75-fold increased risk of delirium in those receiving benzodiazepines (midazolam) (OR 2.75, 95% CI 1.43-5.26, p = 0.002), while associations with opioid use were inconsistent [13]. In a 2006 cohort study of 198 mechanically ventilated ICU patients, lorazepam use was associated with ~20% higher risk of delirium compared to other sedatives [14]. A 2009 RCT comparing dexmedetomidine and midazolam in 366 intubated, mechanically ventilated ICU patients found ~25% reduction in delirium prevalence with dexmedetomidine [15]. While not exhaustive of all the literature, these studies align with our findings that benzodiazepine use increases delirium risk in STICU patients.

Previous psychiatric illness was also a predictor of delirium in our cohort. A population-based cohort study of 1133 cardiac surgery patients in Stockholm, Sweden, looked at depression as a prediction of postoperative delirium and found that 34% of patients who were depressed preoperatively developed delirium as compared with only 24% of the non-depressed patients, an adjusted OR of 2.19 (95% CI 1.43-3.34) [16]. In 2010, a study of 998 non-cardiac surgical inpatients examined independent preoperative factors that might contribute to the development of postoperative delirium. Preoperative depression was a predictor of postoperative delirium (OR = 1.370, 95% CI 1.000-1.876, p = 0.05) and had a more significant impact on postoperative delirium than preoperative education, alcohol use, or pain [17].

In our study, patients receiving dexmedetomidine were more frequently intubated than those in the control group (79.1% vs. 19.3%). Dexmedetomidine sedation was often initiated post-intubation, which accounts for the higher intubation rate in this group. According to the 2013 revision of the “Clinical Practice Guidelines for the Sustained Use of Sedatives and Analgesics in the Critically Ill Adult,” dexmedetomidine is the only sedative approved for non-intubated ICU patients due to its respiratory-sparing properties, allowing its continuation after extubation [6]. Studies have also shown the benefits of dexmedetomidine in intubated patients, including reduced ventilation duration and ICU length of stay compared to other sedatives. A 2015 systematic review found a 22% reduction in ventilation time (95% CI 10%-33%) and a 14% decrease in ICU stay (95% CI 1%-24%) with dexmedetomidine, although evidence quality was low [18]. Further analysis is needed to assess the comparative benefits of dexmedetomidine as ICU care practices evolve.

Although this study adds to the literature, it is not without limitations. As a retrospective single-institution study, it relied on available documentation. We obtained information on TBI and delirium from the assessments, plans, and treatments noted in the EMR, which is only as thorough as the physician who was documenting at the time. There were instances of information tagged from previous notes and of missing or incomplete discharge summaries. This made it cumbersome, and occasionally impossible, to obtain the maximal amount of information for each patient. Additionally, delirium can be defined in more than one way and when a physician noted “altered mental status” with incomplete or short progress notes, it could be difficult to extrapolate if the physician was referring to delirium. In other instances, an ICD code for confusion was documented for a patient, but in the note, the physician would attribute it to TBI. Likewise, there were inconsistencies in completing bedside CAM-ICU assessments, which further complicated diagnosing delirium appropriately. Another limitation was that we did not evaluate the dosing of any of the medications assessed in the study, including dexmedetomidine. Lastly, differences in patient acuity were not evaluated in the study but may have been present based on the differences in intubation rates between the two study groups. However, ISS was comparable between the two groups. While it is possible that intubation may increase the risk of developing delirium, our study did not observe a significant difference in delirium rates between the groups. This finding may further underscore the influence of independent risk factors in the development of delirium.

## Conclusions

Our study further adds to the existing literature on delirium and the use of dexmedetomidine on the prevalence of delirium. Despite the limitations of our study, we found results consistent with the literature that support risk factors for delirium. Application of this data may facilitate the identification of trauma ICU patients at risk for delirium and provide evidence to guide treatment in this population. Future studies should include prospective assessments and evaluate the impact of dosing.

## References

[1] Nelson S, Muzyk AJ, Bucklin MH, Brudney S, Gagliardi JP (2015). Defining the role of dexmedetomidine in the prevention of delirium in the intensive care unit. Biomed Res Int.

[2] Ely EW, Inouye SK, Bernard GR (2001). Delirium in mechanically ventilated patients: validity and reliability of the confusion assessment method for the intensive care unit (CAM-ICU). JAMA.

[3] Sessler CN, Gosnell MS, Grap MJ (2002). The Richmond agitation-sedation scale: validity and reliability in adult intensive care unit patients. Am J Respir Crit Care Med.

[4] Louis C, Godet T, Chanques G, Bourguignon N, Morand D, Pereira B, Constantin JM (2018). Effects of dexmedetomidine on delirium duration of non-intubated ICU patients (4D trial): study protocol for a randomized trial. Trials.

[5] Devlin JW, Skrobik Y, Gélinas C (2018). Clinical practice guidelines for the prevention and management of pain, agitation/sedation, Delirium, Immobility, and sleep disruption in adult patients in the ICU. Crit Care Med.

[6] Barr J, Fraser GL, Puntillo K (2013). Clinical practice guidelines for the management of pain, agitation, and delirium in adult patients in the intensive care unit. Crit Care Med.

[7] Keating GM (2015). Dexmedetomidine: a review of its use for sedation in the intensive care setting. Drugs.

[8] Lee S (2019). Dexmedetomidine: present and future directions. Korean J Anesthesiol.

[9] Ng KT, Shubash CJ, Chong JS (2019). The effect of dexmedetomidine on delirium and agitation in patients in intensive care: systematic review and meta-analysis with trial sequential analysis. Anaesthesia.

[10] Pereira JV, Sanjanwala RM, Mohammed MK, Le ML, Arora RC (2020). Dexmedetomidine versus propofol sedation in reducing delirium among older adults in the ICU: a systematic review and meta-analysis. Eur J Anaesthesiol.

[11] Lewis K, Alshamsi F, Carayannopoulos KL (2022). Dexmedetomidine vs other sedatives in critically ill mechanically ventilated adults: a systematic review and meta-analysis of randomized trials. Intensive Care Med.

[12] Su X, Meng ZT, Wu XH (2016). Dexmedetomidine for prevention of delirium in elderly patients after non-cardiac surgery: a randomised, double-blind, placebo-controlled trial. Lancet.

[13] Pandharipande P, Cotton BA, Shintani A (2008). Prevalence and risk factors for development of delirium in surgical and trauma intensive care unit patients. J Trauma.

[14] Pandharipande P, Shintani A, Peterson J (2006). Lorazepam is an independent risk factor for transitioning to delirium in intensive care unit patients. Anesthesiology.

[15] Riker RR, Shehabi Y, Bokesch PM (2009). Dexmedetomidine vs midazolam for sedation of critically ill patients: a randomized trial. JAMA.

[16] Falk A, Kåhlin J, Nymark C, Hultgren R, Stenman M (2022). Depression is associated with delirium after cardiac surgery-a population-based cohort study. Interact Cardiovasc Thorac Surg.

[17] Smith PJ, Attix DK, Weldon BC, Greene NH, Monk TG (2009). Executive function and depression as independent risk factors for postoperative delirium. Anesthesiology.

[18] Chen K, Lu Z, Xin YC, Cai Y, Chen Y, Pan SM (2015). Alpha-2 agonists for long-term sedation during mechanical ventilation in critically ill patients. Cochrane Database Syst Rev.

